# Dr. George W. Gregory

**Published:** 1915-05

**Authors:** 


**Dr. George W. Gregory,** Albany, 1879, died at his home in Elmira, Feb. 26.

